# Case report: reuse of tirofiban leads to very severe thrombocytopenia

**DOI:** 10.3389/fcvm.2023.1130552

**Published:** 2023-05-19

**Authors:** Yuqing Li, Jiuchun Qiu, Yi Gao, Guangping Li

**Affiliations:** Tianjin Key Laboratory of Logic-Molecular Function of Cardiovascular Disease, Department of Cardiology, Tianjin Institute of Cardiology, the Second Hospital of Tianjin Medical University, Tianjin, China

**Keywords:** tirofiban, thrombocytopenia, severe, anaphylaxis, case report

## Abstract

**Background:**

Telofiban is a class of small molecule non-peptide tyrosine derivatives containing RGD sequences. It is the only platelet surface glycoprotein (GP) IIb/IIIa receptor antagonist (GPI) currently marketed in China. In patients with ST-segment elevation myocardial infarction(STEMI) who receive percutaneous coronary intervention (PCI) with a heavy thrombotic load, postoperative intravenous tirofiban can prevent complications of myocardial ischemia due to sudden coronary artery occlusion. With the increase in the clinical use of tirofiban, the number of adverse reactions related to thrombocytopenia induced by tirofiban has gradually increased. Still, most of them have thrombocytopenia after the first use. We report one case of very severe thrombocytopenia following the reuse of tirofiban.

**Case summary:**

A 65-year-old man of Han nationality, 170 cm in height, 85 kg in weight, and 29.4 BMI, suffered from cerebral infarction 13 years ago and left with right limb movement disorder. Five days before this hospitalization, the patient underwent PCI, and three stents were implanted. After the operation, anti-platelet tirofiban and nadroparin calcium were given, and no thrombocytopenia was found. The patient still retains 80% stenosis due to anterior descending branches and plans to undergo PCI again half a month later. The patient with a history of hypertension, type 2 diabetes, diabetic nephropathy, and cerebral infarction usually took 100 mg of aspirin and 75 mg of clopidogrel, antiplatelet therapy, and had no history of food and drug allergy. One day after discharge, the patient suddenly felt chest tightness and wheezing. The laboratory showed hypersensitivity troponin 2.85 ng/ml (normal 0–0.0268 ng/ml), and the admission ECG showed ST-T changes in leads I, aVL, V5-V6. On the 6th day of hospitalization, PCI was performed, a stent was implanted in the proximal section of the anterior descending branch opening, and tirofiban(10 ug/kg, 3 min bolus, then 0.1 ug/kg/min) antiplatelet therapy was given after surgery. About 10 min after the tirofiban infusion, the patient suddenly shivered, accompanied by convulsions, accompanied by elevated body temperature (up to 39.4°C), accompanied by epistaxis and microscopic hematuria. An urgent blood test showed that the platelets dropped to 1 × 109/L, tirofiban and aspirin stopped immediately, and the antiplatelet therapy of clopidogrel was retained. After infusion of methylprednisolone sodium succinate and gamma globulin, the patient's platelets gradually recovered, and the patient was successfully discharged seven days later in stable condition.

**Conclusion:**

This case is typical of severe thrombocytopenia caused by reusing tirofiban. This case may provide new insights into: 1. Patients who did not have thrombocytopenia after the first use of tirofiban may still have extremely severe thrombocytopenia after re-exposure to tirofiban. Routine platelet count monitoring and early identification of thrombocytopenia are the essential links. 2. Thrombocytopenia caused by re-exposure to tirofiban may have a faster onset, deeper degree, and slower recovery due to antibodies retained after the first exposure to tirofiban; 3. Platelet transfusions may not be necessary for patients with severe thrombocytopenia; 4. Immunosuppressants help suppress the body's immune response, promote platelet recovery, and can be reduced or discontinued when platelets rise and may be safe; 5. After tirofiban for PCI, continuing the maintenance dose of clopidogrel may be safe if the patient has no significant bleeding events.

## Introduction

Coronary angiography (CAG) and PCI have become standard methods for evaluating and treating coronary artery lesions. Patients undergoing CAG routinely take antiplatelet and anticoagulant drugs (1). Platelet aggregation inhibitors have better clinical benefits in patients at high risk of coronary artery disease with a heavy thrombus burden who undergo emergency PCI (2). As the only GPI currently marketed in China, tirofiban reduces the risk of thrombosis by inhibiting platelet aggregation. However, tirofiban can cause severe thrombocytopenia in rare cases (3).

The glycoprotein IIb/IIIa receptor aggregates platelet by linking the vWf factor and fibrinogen. As a small non-peptide tyrosine derivative, tirofiban competes for glycoprotein IIb/IIIa receptors. When administered intravenously, platelet aggregation is inhibited in a concentration-dependent manner (4). Current studies have found that typical GPI-induced thrombocytopenia is mainly divided into five modes: (1) Occurs within 12 h after the first contact; (2) Within 12 h of the second contact; (3) Delayed thrombocytopenia: 5–7 days after treatment; (4) Pseudothrombocytopenia: platelet aggregation; (5) Allergic reactions secondary after exposure (5). With the widespread clinical use of tirofiban, reports of thrombocytopenia have gradually increased (6). Most cases are thrombocytopenia after the first exposure, and thrombocytopenia due to the second exposure is rare.

Here, we report a very severe thrombocytopenia caused by reusing tirofiban.

Timeline: See Table 1.

**Table 1:** Timeline of major events before and after the patient's hospitalization.

5-Days before	Coronary angiography was performed, a total of 3 stents were implanted, and 80% of the lesions presentation were opened on a later date before retention. Postoperative application of tirofiban and calcium natracalcin showed no thrombocytopenia
Day 0	A 65-year-old male patient, emergency laboratory test: hypersensitive troponin 2.85 ng/ml, admitted ECG: I, AVL, V5-V6 LEAD ST-T changes, again due to severe chest tightness and asthma admitted to the cardiovascular department of our hospital
Day 1	Initially considering non-ST-segment elevation myocardial infarction(N-STEMI), aspirin and clopidogrel dual antiplatelet therapy, and natreparin calcium anticoagulation therapy, no obvious thrombocytopenia was seen
Day 6	Coronary angiography was performed before opening the descending branch and implanted with one stent, and after surgery, ticrofiban was given symptomatic anti-plate therapy. After 10 min of tirofiban infusion, the patient suddenly shivered, accompanied by high fever and sweating, and the emergency blood routine showed that platelets dropped to 1 × 109/L. Discontinue all anticoagulant and antiplateau drugs and retain clopidogrel alone
Day 7–10	The patient had scattered ecchymosis with subcutaneous bleeding points, microscopic hematuria, and epistaxis, and methylprednisolone sodium succinate 40 mg bid for 4 days and gamma globulin 10 g/d for 2 days. On the 10th day of admission, platelets were rechecked and recovered to 18 × 109/L
Day 13	The ecchymosis gradually subsided, and no bleeding spots were visible to the naked eye. The platelets recovered to 84 × 109/L and the patient recovered and was discharged from the hospital
1-Month post-discharge	The patient's platelets returned to normal

## Case presentation

**Figure 1:**
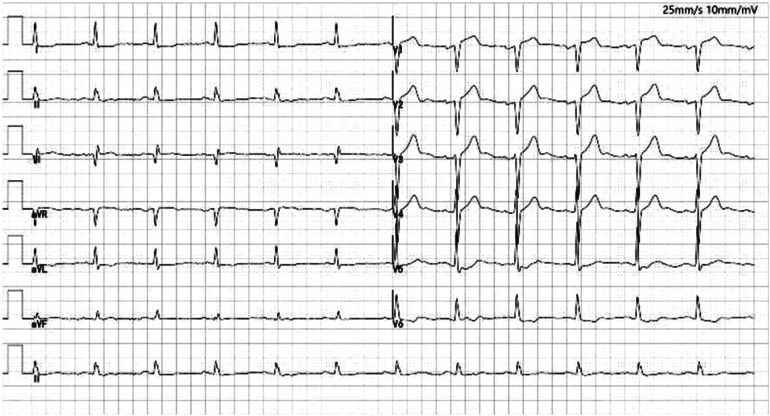
Electrocardiogram of the patient with I, AVL, V5-V6 lead ST-T changes. There is pathological Q waves (lead III, V1-V2). Poor increase of R waves in thoracic leads.

A 65-year-old man of Han nationality, 170 cm in height, 85 kg in weight, and 29.4 BMI, suffered from cerebral infarction 13 years ago and left with right limb movement disorder. Coronary angiography performed one year before hospitalization showed three-vessel disease, and no stent was implanted. The patient underwent CAG five days before hospitalization, three stents were planted during the operation, and 80% of the lesions with the preservation of the anterior descending branch were opened later. The patient had a history of hypertension, type 2 diabetes mellitus, diabetic nephropathy, and cerebral infarction and had no history of food or drug allergy or thrombocytopenia. After hospitalization, the laboratory showed that hypersensitivity troponin was 2.85 ng/ml. The ECG on admission showed ST-T changes in leads I, aVL, V5-V6 (Figure 1), and non-ST-segment elevation myocardial infarction(NSTEMI) was initially considered. Blood tests did not show apparent thrombocytopenia. Cardiac examination reveals no prominent murmurs, friction rubs, or galloping rhythms.

On the 6th day after admission, the patient was admitted to the cardiac catheterization laboratory for PCI. During the operation, 80% stenosis of the proximal section of the anterior descending branch opening was found, the occlusive lesion was opened, one stent was implanted, and the patient returned to the ward after the operation. Because of the severe thrombus burden of the patient, tirofiban (10 ug/kg, 3 minute bolus, followed by 0.1 ug/kg/min [the guidelines recommend the dose] (7)) instillation was started. Ten minutes after the tirofiban infusion, the patient suddenly shivered, accompanied by a continuous increase in body temperature, up to 39.4°C, with heavy sweating. It was considered that the patient had a high possibility of sudden hypersensitivity reaction, and the allergen was not known, so transient bacteremia and infusion reaction could not be ruled out. Blood routine examinations and blood cultures were taken urgently. Diphenhydramine hydrochloride injection (1 ml; 20 mg) by intramuscular injection for symptomatic anti-allergic treatment. It was found that the platelet count decreased to 1 × 10^9^/L, but the blood was drawn again to exclude the test error, and the reexamination was still 1 × 10^9^/L. The laboratory physician performed a blood-smear examination of the peripheral-blood sample, confirming a profound platelet deficiency with no platelet aggregation. The smear results were reported to the attending physician.

**Figure 2:**
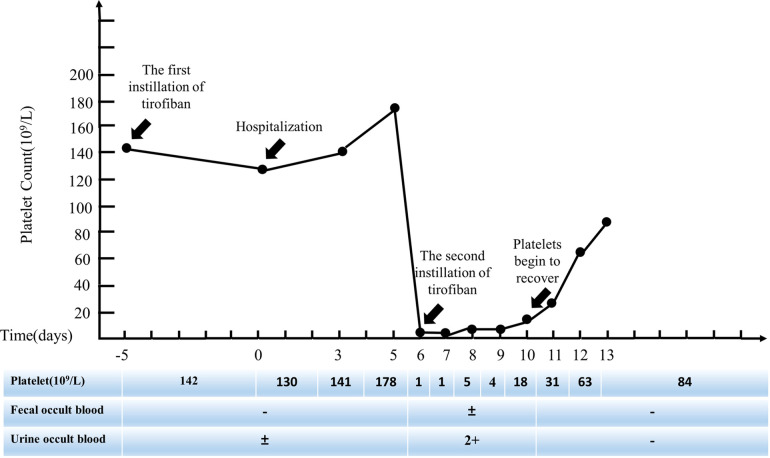
Evolution of the platelet count (days before and after stopping tirofiban infusion).

All antiplatelet and anticoagulant drugs were discontinued immediately. Given the patient's severe thrombotic burden, clopidogrel antiplatelet therapy was still retained. On the 7th day after hospitalization, the patient gradually developed scattered ecchymosis with subcutaneous bleeding spots, microscopic hematuria, and epistaxis. Other standard laboratory tests include prothrombin time (PT), partial thromboplastin time (PTT), fibrinogen, and D-dimer. Haptoglobin, reticulocyte count, and lactate dehydrogenase (LDH) are also expected. Methylprednisolone sodium succinate 80 mg for four days and gamma globulin 200 mg/kg/day for two days for symptomatic anti-inflammatory treatment, daily blood routine (Figure 2), and occult blood tests, including urine and stool samples. On the 10th day of hospitalization, the patient's platelets recovered to 18 × 10^9^/L. On the 13th day of hospitalization, the ecchymosis throughout the patient's body gradually subsided, and no visible bleeding points were seen. The re-examination showed that platelets recovered to 84 × 10^9^/L, and the patient was discharged. One month after discharge, the patient's repeated blood routine showed that platelets had returned to normal.

## Discussion

Tirofiban-induced thrombocytopenia(TIT) gradually increases in clinical practice with the widespread use of tirofiban, and the severity usually ranges from mild to severe. Classification according to the degree of thrombocytopenia: mild (50–99 × 10^9^/L), severe (20–49 × 10^9^/L), very severe (20 × 10^9^/L). Very severe thrombocytopenia (Plt < 20 × 10^9^/L) is rare and should be considered an emergency, usually requiring platelet transfusion. In some reported cases, patients developed thrombocytopenia with symptoms such as chills, fever, and hypotension (8). Tirofiban has a half-life of approximately 2 h and a rapid onset of action. For TIT, platelet count generally recovered 2.1 days after drug withdrawal. According to the current reports, the incidence of severe thrombocytopenia (Plt<<50 × 10^9^/L) ranges from 0.2% to 0.5% of tirofiban (9). The combination of GPI and PCI has been shown to reduce mortality after revascularization in patients with STEMI and NSTEMI (10). Thrombocytopenia is one of the main adverse effects of tirofiban, but thrombocytopenia has primarily been reported after initial application. Rapid thrombocytopenia with re-exposure to tirofiban has only been reported in a few cases (3, 6).

Drug-mediated immune thrombocytopenia (DITP) is a spontaneous immune-mediated response in which anti-platelet antibodies generally appear 1–2 weeks after using a new drug or re-exposure to a drug after a previous exposure history. TIT belongs to a species of DITP. At present, it is believed that the mechanism of tirofiban-induced thrombocytopenia is roughly divided into two types: one is the antigen-antibody reaction in the patient's body, resulting in platelets being cleared by the body's immune system (11); The second is that tirofiban induces the conformational change of glycoprotein receptor on the platelet surface, and the new antigenic determinants are produced and recognized by the liver and eventually eliminated (12). Acute platelet destruction after GPI use suggests that non-immune factors may play a role. Still, recent studies have confirmed that drug-dependent antibodies may be the leading cause of platelet destruction (8). The detection of antibodies recognizing the GPIIb/IIIa site in the blood of patients with thrombocytopenia further verified the possibility of immune factors. However, there is still no precise mechanism to explain the cause of thrombocytopenia (11).

**Figure 3:**
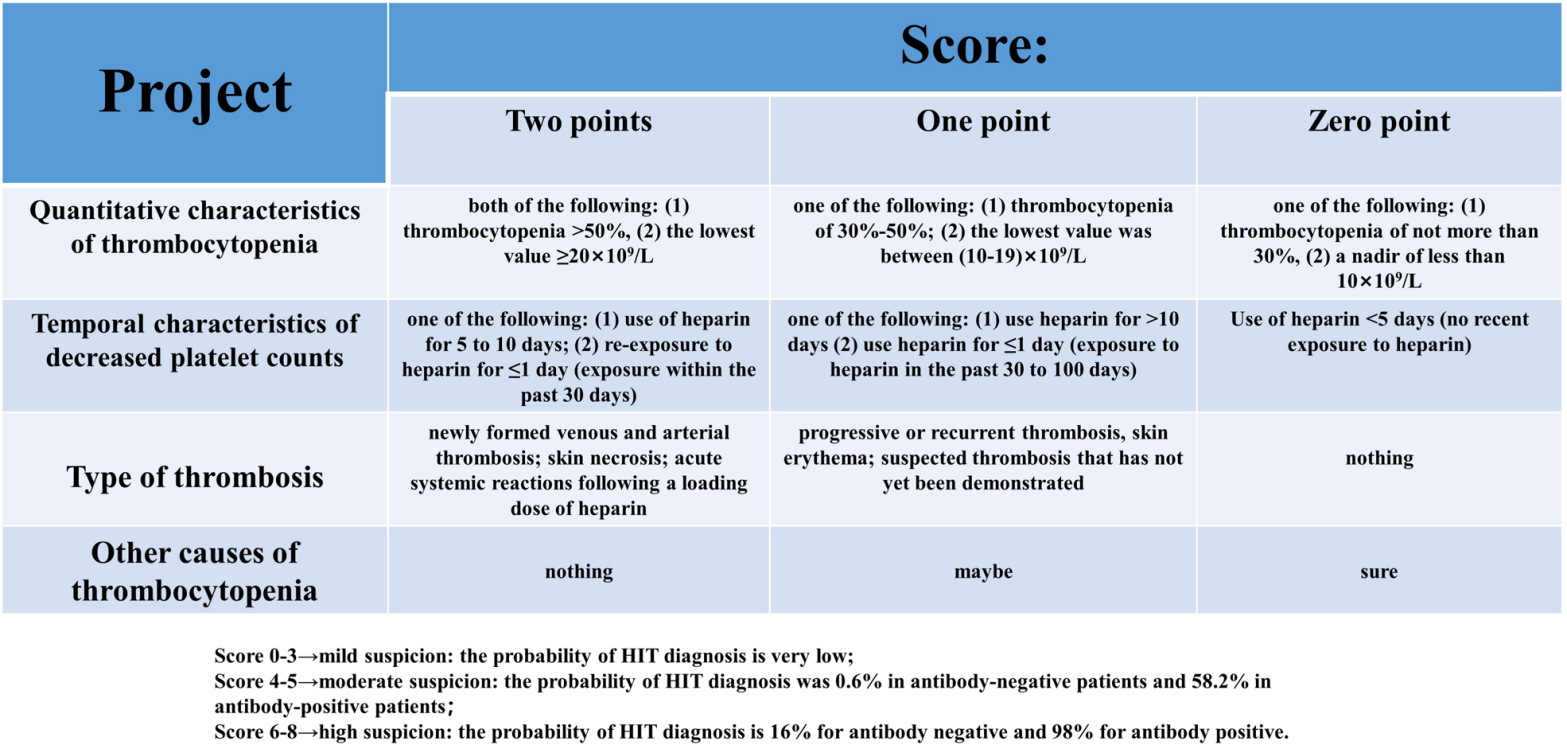
Scoring system for heparin-induced thrombocytopenia (H IT 4T's score).

Like other DITPs, TIT is also an exclusive diagnosis and other drug-induced thrombocytopenia needs to be excluded from making a definite diagnosis. In addition to intravenous tirofiban infusion after PCI, the patient received intravenous nadroparin calcium 4000 IU during the whole treatment and long-term oral antiplatelet therapy with aspirin 100 mg and clopidogrel 75 mg before CAG. These factors could not be excluded from participating in the patient's thrombocytopenia. Pseudo-thrombocytopenia due to laboratory testing was also included as one of the leading differential diagnoses. Pseudo-thrombocytopenia usually refers to using the anticoagulant EDTA in sample tubes that may cause platelets to clump, resulting in artificially low platelet counts. The diagnosis of pseudo-thrombocytopenia can be excluded by performing a peripheral blood smear without platelet aggregation and repeating the platelet count (13). HIT is caused by a combination of heparin and platelet factor 4 (PF4) autoantibodies, and platelets are widely activated, eventually leading to thrombosis and thrombocytopenia. The “4Ts score (14)"(Figure 3) is a pre-test scoring system for HIT designed to play a suggestive role in the clinical diagnosis of HIT. The “4Ts score” has a high sensitivity and negative predictive value for diagnosing HIT. HIT can be ruled out in patients with low clinical likelihood, and HIT-antibody testing and continuous platelet count monitoring are unnecessary. HIT is divided into two types. Type I mainly occurs within five days after the application of heparin and is related to the direct activation of platelets by heparin. Most of them are transient platelet decline, to a lesser extent, and are non-immune thrombocytopenia. Type II occurs 5 to 15 days after heparin application, is associated with PF4 and heparin forming heparin/PF4 complexes, and is an immune response with thrombosis rather than bleeding. The patient had been exposed to heparin for coronary angiography one year before and for the first PCI five days before hospitalization, and no thrombocytopenia was observed. The lowest platelet count was 1 × 10^9^/L. No clinical symptoms related to thromboembolism were found. The final “4Ts score” was only two, indicating that the probability of HIT was very low and HIT could be ruled out (15). The previous “CAPRIE” study (16) showed that aspirin and clopidogrel had a good effect on the prognosis of CAG patients, and thrombocytopenia was rare in both groups. Although very rare, clopidogrel can also cause DITP, and the mechanism is currently believed to be the production of IgG-type autoantibodies against ADAMTS13 induced by the drug (17). So far, no extremely severe thrombocytopenia caused by aspirin or clopidogrel alone has been reported. Long-term antiplatelet therapy with aspirin and clopidogrel was initiated one year earlier, and thrombocytopenia was not observed, so this diagnosis can be ruled out. Based on the clinical presentation, laboratory tests, and timeline of events during hospitalization, thrombocytopenia in patients is likely due to immune thrombocytopenia mediated after reusing tirofiban.

**Figure 4:**
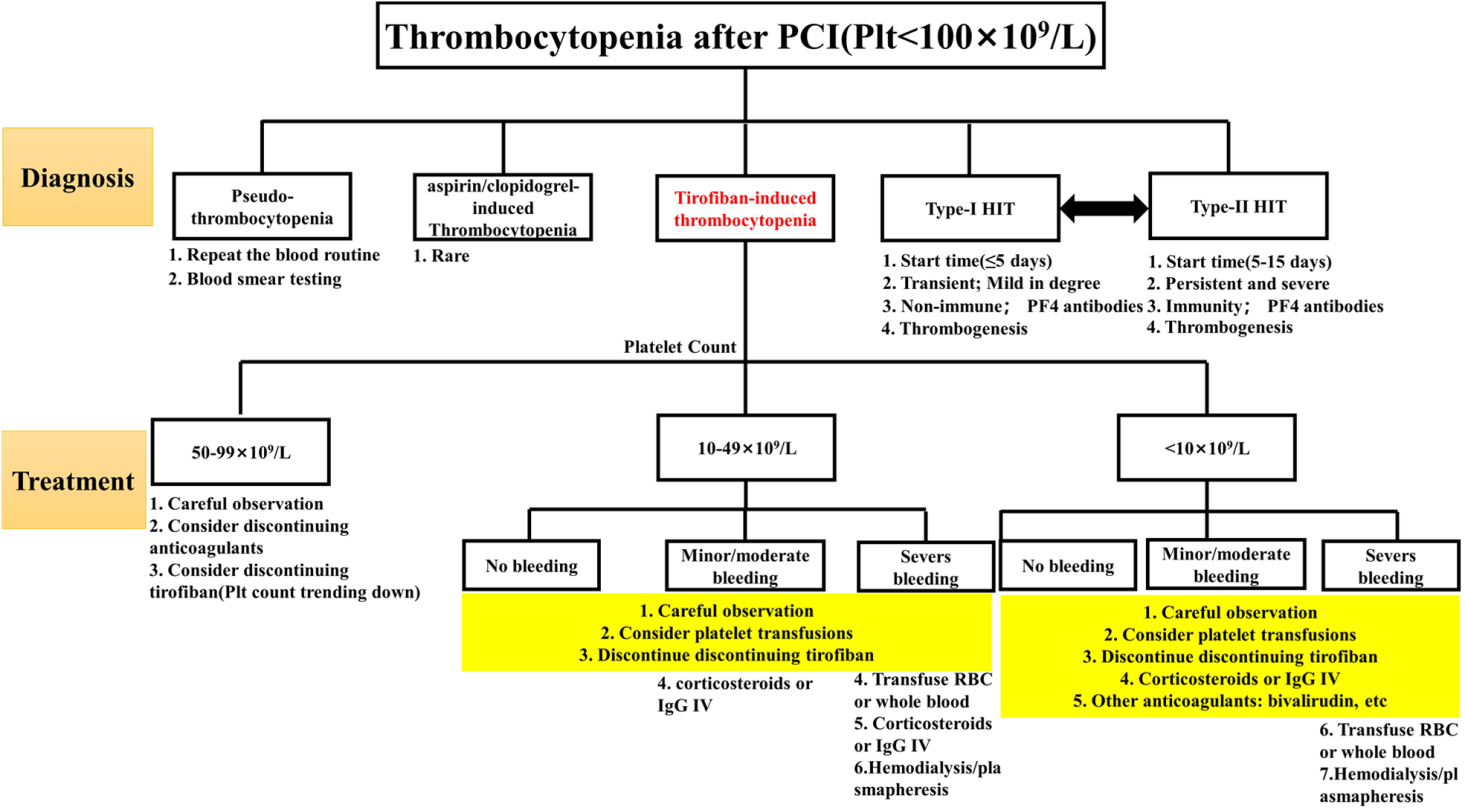
Differential diagnosis and treatment of tirofiban-induced thrombocytopenia.

As a kind of DITP, TIT follows the general treatment principles of DITP. Still, its treatment should be adjusted according to the degree of thrombocytopenia and bleeding, even if the degree of thrombocytopenia is not necessarily proportional to the degree of bleeding. The details were as follows: (1) Patients with platelet count decreased to 50–99 × 10^9^/L were generally maintained under observation, and tirofiban infusion would be stopped if the platelet count decreased continuously; (2) Patients whose platelet count decreased to less than 50 × 10^9^/L. There is a strong rationale for first withholding tirofiban in treating very severe thrombocytopenia since tirofiban is cleared from the circulation within one hour after discontinuation (18). For TIT, corticosteroid or immunoglobulin infusion may be considered without long-term platelet recovery. Unfortunately, there is no clinical trial or basic research to confirm that corticosteroids and immunoglobulin play a decisive role in promoting platelet recovery. More empirical treatment exists in clinical reports (19, 20). Switching to another antiplatelet agent, such as bivalirudin, may be considered if the patient is at high risk for thrombosis. Bivalirudin has fewer bleeding events and 30-day net adverse clinical events than tirofiban (21); (3) For patients with thrombocytopenia <10 × 10^9^/L and patients with severe bleeding, it is recommended to stop all anticoagulants and antiplatelet drugs. Platelet transfusion is recommended (22). For life-threatening bleeding (brain, lung, or pericardium), hemodialysis or plasmapheresis may facilitate the rapid metabolism of tirofiban in the body. For details, see Figure 4. On the one hand, considering the patient's economic factors, a half-dose of gamma globulin (200 mg/kg/d) combined with methylprednisolone sodium succinate (80 mg/d) was used for anti-immune treatment, and the drug was stopped in time when the patient's platelet showed a recovery trend. On the other hand, clopidogrel antiplatelet therapy has been retained to prevent the risk of thrombosis caused by the rapid rise of platelets. Ultimately, the patient did not have prominent bleeding events, and the platelet gradually returned to normal.

Thrombocytopenia due to this case is rare compared with patients with thrombocytopenia after the first exposure to tirofiban. The onset of the disease is shorter (10 min after administration; The standard time is from a few hours to seven days after starting the infusion), symptoms are more typical at the beginning (platelets drop to 1 × 10^9^/L), Platelet recovery time is longer (platelet levels do not recover until about one week after discontinuation of tirofiban, much longer than the half-life of tirofiban by two hours). Therefore, it is essential to design a concise and straightforward procedure to prevent the occurrence of highly severe TIT. (1) Screening high-risk patients before tirofiban administration: the PRISM-PLUS study found that older age, lower body weight, female gender, and limited creatinine clearance (<30 ml/min) were associated with a higher risk of bleeding (23). Not coincidentally, Yi et al. designed a clinical preoperative risk model by reviewing the clinical features of patients with tirofiban-induced thrombocytopenia, identifying five independent risk factors: age ≥ 65 years, white blood cell ≥ 12 × 10^9^/L, diabetes, congestive heart failure, and chronic kidney disease. Applying preoperative risk models allows us to assess the risk of thrombocytopenia in patients and then adopt instillation dose reduction or alternative drug methods to improve patient safety (24); (2) Selection of infusion dose during the application of tirofiban: Wang et al. conducted a safety study on different amounts of tirofiban instillation, showing that high-dose tirofiban instillation was associated with a significant reduction in the incidence of major cardiac adverse events but was accompanied by a higher bleeding rate and thrombocytopenia. It suggests that the dose of tirofiban should be adjusted reasonably according to the patient's condition and bleeding risk (25); (3) Routine monitoring after tirofiban application: routine blood tests should be rechecked at two, six, twelve, and twenty-four hours after tirofiban application, and regularly rechecked once a day after tirofiban withdrawal until discharge to prevent failure to detect thrombocytopenia in time (19, 20); (4) Early identification after TIT: when the patient's blood routine shows extremely severe thrombocytopenia, it is essential to analyze the related drugs that may cause thrombocytopenia in the patient, conduct related laboratory tests to confirm/exclude the diagnosis, and finally determine the causative drug.

## Data Availability

The raw data supporting the conclusions of this article will be made available by the authors, without undue reservation.
